# Supplementary material for: Differential Effects of D9 Tetrahydrocannabinol (THC)- and Cannabidiol (CBD)-Based Cannabinoid Treatments on Macrophage Immune Function In Vitro and on Gastrointestinal Inflammation in a Murine Model
Source: Biomedicines. 2022 Jul 26;10(8):1793. doi: 10.3390/biomedicines10081793 (PMC9332744; doi:10.3390/biomedicines10081793)
Supplement: Supplementary file 1 [file biomedicines-10-01793-s001.zip › biomedicines-1783629-supplementary.pdf]

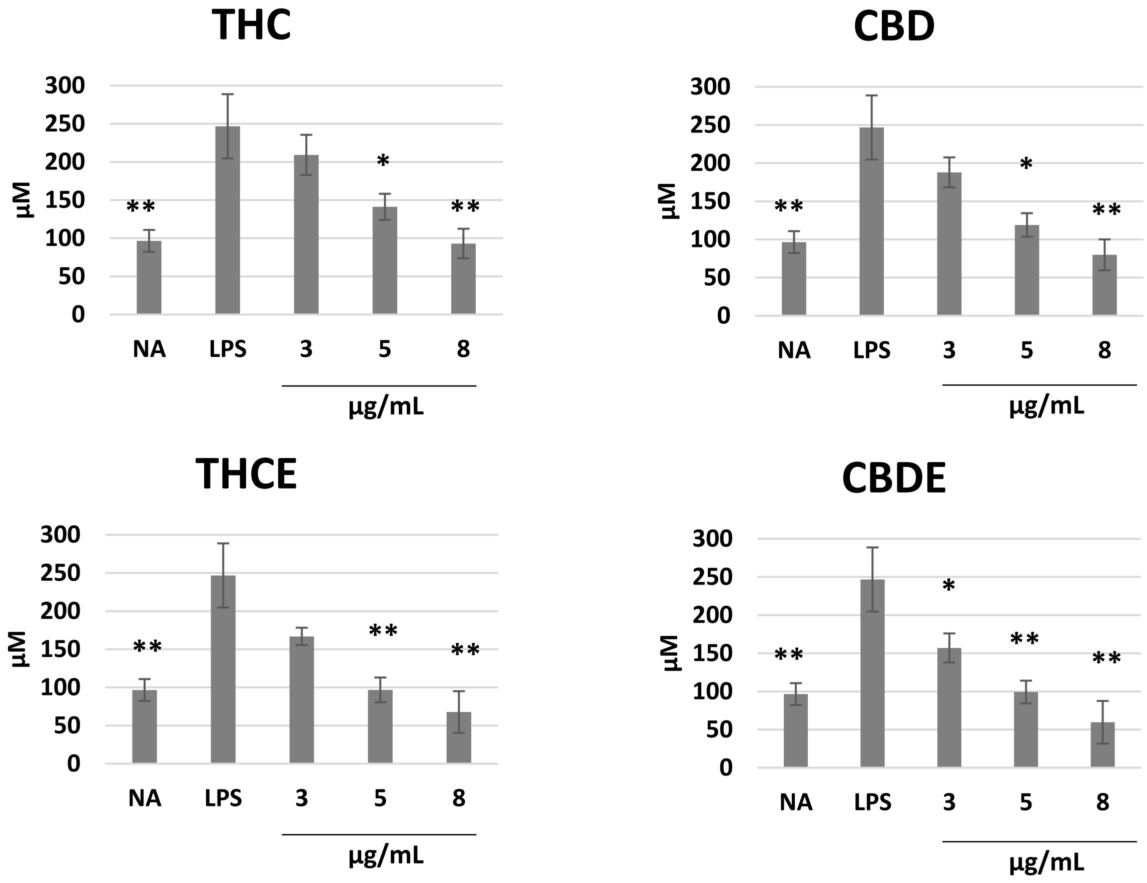

Figure S1. Dose dependent inhibition of nitric oxide production by peritoneal macrophages treated with pure CBD/THC or cannabis extracts. Peritoneal macrophages from C57bl/6 female mice were activated for 24h with LPS, in the presence of cannabinoid treatments at the indicated concentrations. *n* = 5 mice. . *p* value as compare to activated control cells *,< 0.05; **,< 0.01; NA- non-activated LPS – lipopolysaccharide activated macrophages, THC- D9 tetrahydrocannabinol , CBD- cannabidiol , THCE- high THC cannabis extract, CBDE- high CBD cannabis extract

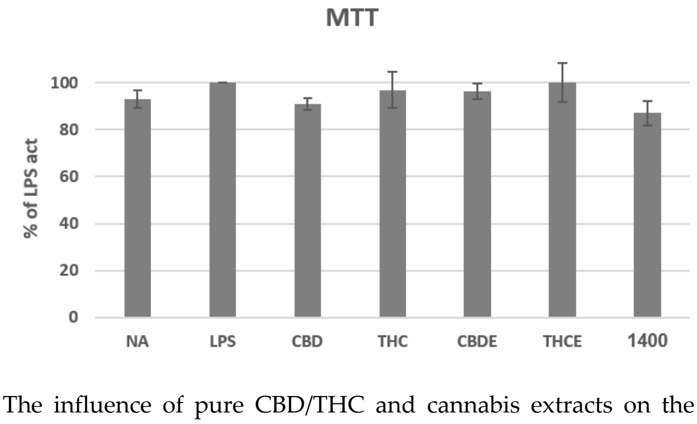

Figure S2. The influence of pure CBD/THC and cannabis extracts on the viability of LPS activated peritoneal macrophages. Peritoneal macrophages from female C57bl/6 mice were activated for 24h with

LPS, in the presence of cannabinoid treatments. 1400, a specific iNOS inhibitor, served as control. Cell viability was analyzed using MTT assay. NA- non-activated LPS – lipopolysaccharide activated macrophages, THC- D9 tetrahydrocannabinol , CBD- cannabidiol , THCE- high THC cannabis extract, CBDE- high CBD cannabis extract

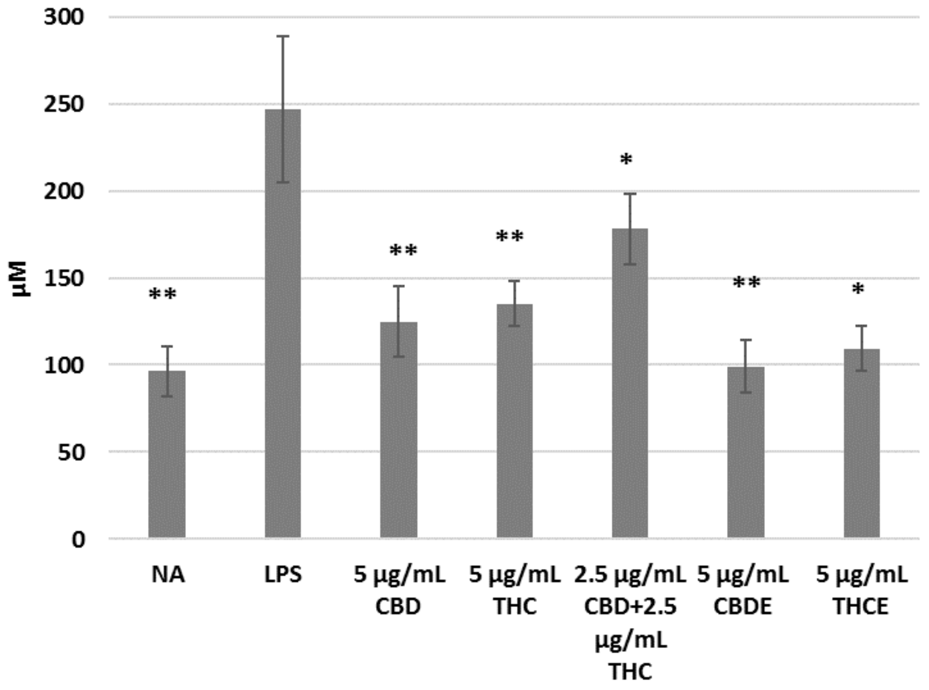

Figure S3. Combination of THC and CBD (2.5 µg/mL of each) is less effective than the pure cannabinoids. Peritoneal macrophages from C57bl/6 female mice were activated for 24h with LPS, in the presence of cannabinoid treatments at the indicated concentrations. $n = 5$ mice. . p value as compare to activated control cells *,< 0.05; **,< 0.01; NA- non-activated LPS – lipopolysaccharide activated macrophages, THC- D9 tetrahydrocannabinol , CBD- cannabidiol , THCE- high THC cannabis extract, CBDE- high CBD cannabis extract

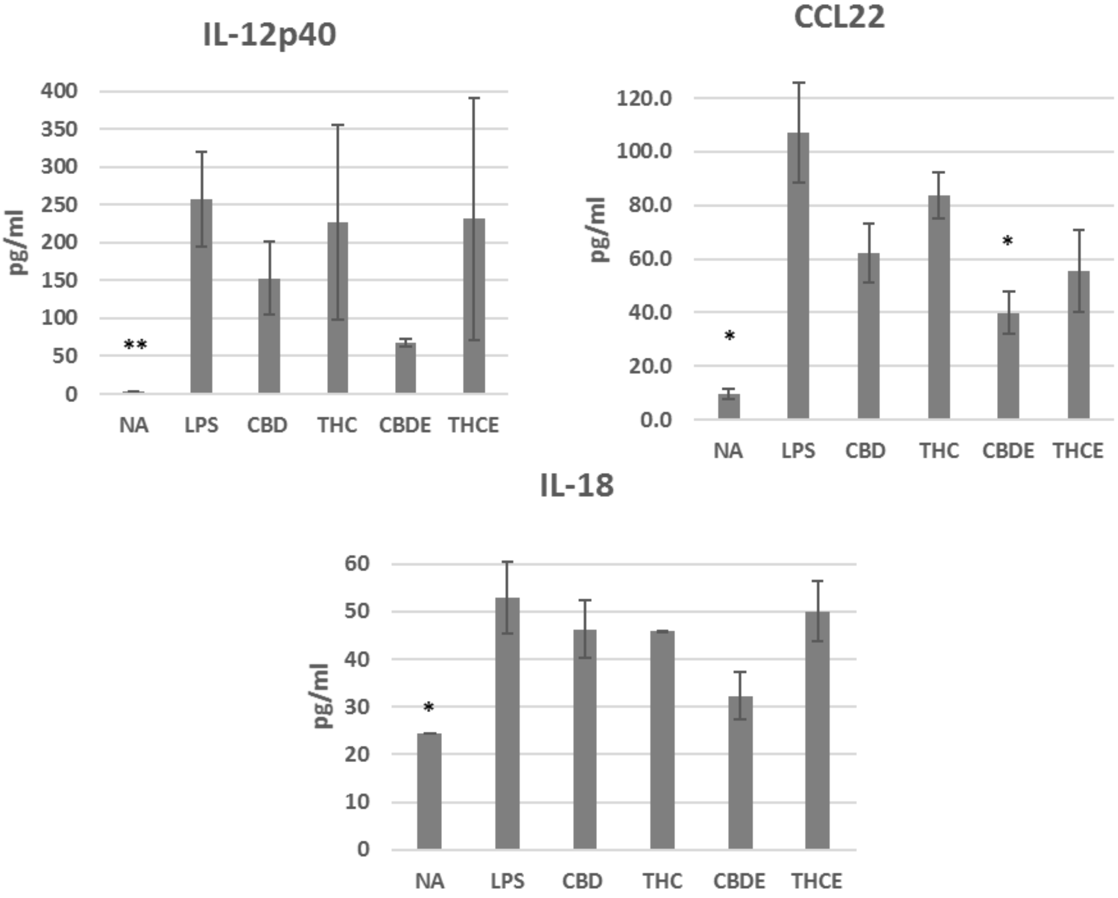

Figure S4. The influence of pure CBD/THC and cannabis extracts on cytokine/chemokine production of LPS activated peritoneal macrophages. Peritoneal macrophages were activated for 24h with LPS, in the presence of cannabinoid treatments, as in Figure 1. *n* = 3 mice. IL12p40, CCL22, and IL18 levels in the culture supernatant were detected. Results are expressed as mean +SEM. *p* value as compare to activated control cells *, < 0.05; **, < 0.01; NA- non-activated LPS – lipopolysaccharide activated macrophages, THC- D9 tetrahydrocannabinol , CBD- cannabidiol , THCE- high THC cannabis extract, CBDE- high CBD cannabis extract
